# Plectin in Skin Fragility Disorders

**DOI:** 10.3390/cells10102738

**Published:** 2021-10-14

**Authors:** Dimitra Kiritsi, Leonidas Tsakiris, Franziska Schauer

**Affiliations:** 1 Department of Dermatology, Faculty of Medicine, Medical Center-University of Freiburg, 79104 Freiburg, Germany; franziska.schauer@uniklinik-freiburg.de; 2 MediClin Herzzentrum Lahr/Baden, 77933 Lahr, Germany; tsakirisl@msn.com

**Keywords:** epidermolysis bullosa, paraneoplastic pemphigus, hemidesmosome, intermediate filaments, plakin, skin blistering, muscular dystrophy, pyloric atresia

## Abstract

Plectin is a multi-faceted, 500 kDa-large protein, which due to its expression in different isoforms and distinct organs acts diversely as a cytoskeletal crosslinker and signaling scaffold. It functions as a mediator of keratinocyte mechanical stability in the skin, primarily through linking intermediate filaments to hemidesmosomes. Skin fragility may occur through the presence of mutations in the gene encoding for plectin, *PLEC*, or through the presence of autoantibodies against the molecule. Below, we review the cutaneous manifestations of plectinopathies as well as their systemic involvement in specific disease subtypes. We summarize the known roles of plectin in keratinocytes and fibroblasts and provide an outlook on future perspectives for plectin-associated skin disorders.

## 1. Introduction

Plectin, a member of the plakin family, is a 500 kDa-large protein with various roles as a cytoskeletal crosslinker and signaling scaffold. Twelve isoforms of the molecule have been described, arising through alternative splicing, primarily within the N-terminal domain [1]. Plectin is a multi-faceted, highly interesting molecule since different isotypes are expressed in various cells (including keratinocytes, fibroblasts, myoblasts, and Schwann cells), fulfilling distinct functions [1]. Only four are expressed in the epidermis, namely P1, P1a, P1c, and P1f [1]. Structurally, plectin has a central α-helical coiled-coil rod domain with N- and C-terminal globular domains [2]; its role in the skin and other organs is revealed by the effects of mutations in the distinct plectin domains and isoforms [3]. In the skin, plectin is a mediator of keratinocyte mechanical stability, primarily through the connection of intermediate filaments (IFs) to hemidesmosomes (HD) [4]. Specifically, the N-terminus contains an actin-binding domain and a plakin domain with binding sites for integrin α6β4 [5,6] and collagen XVII/ BP180 [7], while the C-terminus comprises six plakin domains with binding sites for IFs, vinculin, and integrins [8,9,10]. Plectin is also a component of desmosomes and focal adhesions [11]. It has been suggested that the binding of plectin to integrin β4 prevails over that with the actin cytoskeleton, resulting in the preferential distribution of plectin in the hemidesmosomes [12], which accounts for the important role of plectin in regulating keratinocyte adhesion. It also plays a role in organizing mitochondria and nuclei within the intermediate filament network [11,13], thus being involved in the cellular metabolome [14]. Skin fragility may occur through the presence of mutations in the gene encoding for plectin, *PLEC*, or through the presence of autoantibodies directed against the molecule. To date, more than 100 mutations have been reported to result in different inherited disease subtypes (Human Gene Mutation Database- HGMD 2021.1). The manifestations of these specific disorders are summarized below.

## 2. Plectin in Hereditary Skin Fragility Disorders

**Figure 1:**
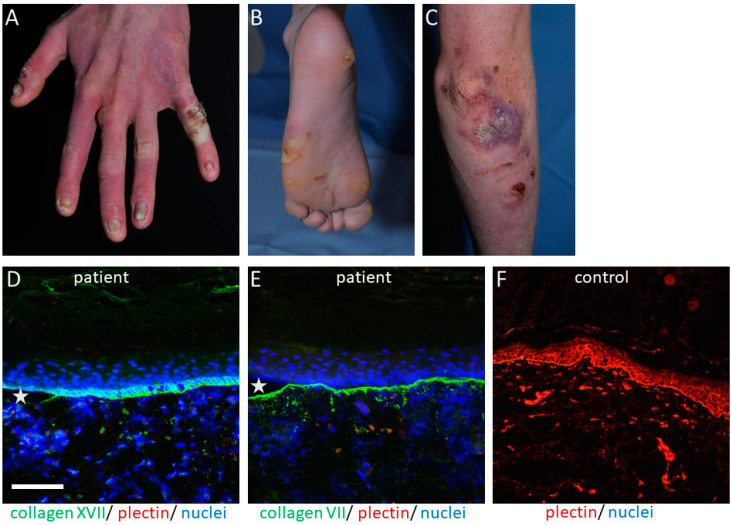
Cutaneous manifestations and IF findings in a 20-year-old patient with EBS-MS: Firm blisters and erosions on hands (**A**), feet (**B**), and elbow (**C**), together with crusts, are found on the skin of a patient with strongly reduced plectin expression. Hypopigmented patches, as well as erythematous macules, developed after healing of the blisters, as well as focal palmoplantar keratoderma, mostly on the mechanically stressed areas, causing great discomfort to the patient. (**D**) Collagen XVII (green, (**D**)) was stained on both the blister roof and floor, while (**E**) collagen VII only stained on the floor of the patient’s skin (20× magnification), thus providing a “pseudojunctional EBS” immunofluorescence pattern. Plectin (red in (**D**,**E**)) is almost completely absent, compared to the control skin (**F**). The nuclei are visualized with DAPI, with asterisks highlighting the blisters.

The *PLEC* gene is a huge gene on chromosome 8q24, consisting of 32 exons. Mutations have mostly been disclosed within exons 31 and 32 and cause the skin fragility disorder epidermolysis bullosa simplex (EBS) (Figure 1A–C). On the tissue level, plectin deficiency results in a split within the keratinocytes of the basal layer of the epidermis, and although considered exceedingly rare, around 8% of patients with EBS carry mutations in the *PLEC* gene [15]. Since blisters are within the basal keratinocytes directly above the HD, only fragments of the destroyed HD exist on the blister floor, and thus old blisters frequently appear to be junctional (also called “pseudojunctional” EBS) (Figure 1D,E) [16,17]. Electron microscopy reveals hypoplastic HD, with normal extracellular portions but a poor inner plaque assembly [16,18]. A variety of antibodies have been described to specifically detect different plectin domains, which has proven helpful for the prognostication of patients’ disease severity, as explained below.

The disease shows a great phenotypic variability [19], which is only partially explained by the presence or not of a residual plectin expression (Table 1). Individuals with a complete loss of plectin due to loss-of-function mutations suffer from either the most severe subtype, EBS with pyloric atresia (EBS-PA), or EBS with muscular dystrophy (EBS-MD). A retrospective study proposed, as a general rule, that loss-of-function mutations in exon 31 accounted for cases with EBS-MD, whereas EBS-PA was caused by mutations outside exon 31 [20], with some exceptions [21,22]. The alternative splicing of exon 31, resulting in a rodless but still partially functional plectin form, was suggested to account for the milder phenotype [20,23]. Notably, patients with the same *PLEC* mutations might have a different phenotype; thus, other genetic, epigenetic, or environmental factors could drive the specific disease manifestations [22,24].

The clinical hallmark of the disease is the life-long, trauma-induced blistering of skin and mucosa. The EBS-PA subtype is characterized by congenital pyloric atresia and severe skin fragility, with areas of aplasia cutis, and was originally thought to be associated with an early demise [25]. However, a few cases have been reported where the patients survived the neonatal period, most of them developing muscular dystrophy and, interestingly, urological abnormalities [22,26,27].

Most patients with EBS-MD show blisters and erosions at birth or within childhood, which are often hemorrhagic and develop in mechanically stressed skin [22]. These heal with residual skin atrophy with no scarring. Recurrently, firm blisters on hands and feet typically impair patients’ mobility and quality of life (Figure 1A–C). Palmoplantar keratoderma is also frequently present [26]. Onychodystrophy or nail loss occurs, as well as enamel defects. The involvement of mucous membranes is characteristic, especially of the oral mucosa, but also laryngotracheal and gastrointestinal ones [22]. In a few cases, diffuse alopecia has been reported in adulthood [27,28]. Hypertrophic hypergranulation with subsequent scarring and adhesions of the vocal folds, resulting in stridor and a hoarse voice, seems to occur in a few cases, which has been similarly described in laryngo-onycho-cutaneous syndrome [18,29,30].

Muscular dystrophy is of a delayed progressive type and can occur at different ages, ranging from infancy to late adulthood [26], with variable severity. It results from desmin protein aggregates within the skeletal muscles [31], disorganizing the myofibrillar apparatus and evoking an increased chaperone expression [32]. In addition, the cytoskeletal abnormalities are linked to mitochondrial anomalies [31], which also contribute to progressive muscle damage. Typically, individuals with EBS-MD suffer from slowly advancing muscle weakness, while dilated cardiomyopathy or ventricular hypertrophy might also develop. In some cases, ophthalmoplegia and ptosis have been reported, as well as cerebral atrophy [29]. A distinct subtype of the disease with features of limb-girdle muscular dystrophy type 2Q without any cutaneous abnormalities is connected to mutations in exon 1f of the *PLEC* gene [11,30].

A probably frequently unrecognized manifestation of plectinopathies is the development of dilated cardiomyopathy. Plectin is expressed in cardiac muscle [31,33], while *PLEC* mutations seem to result in a perturbed co-localization of plectin and desmin in the myocardium. The recognition of cardiomyopathy is challenging since, in the initial stages, the symptoms are rather mild and activity-dependent, which is already reduced in patients with EBS-MD [33]. In later stages, respiratory problems become more prominent and frequently represent the reason for an early demise, but unrecognized cardiomyopathy might deteriorate prognosis. Thus, cardiological investigations should be organized early on in patients with plectinopathies. Follow-ups have to be performed on an at least bi-yearly basis initially and, if features of cardiomyopathy are present every six months, accompanied by MRI imaging. Furthermore, in one patient, left ventricular hypertrabeculation/noncompaction was diagnosed [34], which might be associated with malignant arrhythmias; thus, besides echocardiography, electrocardiographic investigations should also be considered in EBS-MD patients. This is in line with the identification of a missense *PLEC* variant associated with an increased risk of atrial fibrillation [35]. However, in a study of 359 international patients, no sufficient indication of the role of *PLEC* variants in arrhythmogenic right ventricular cardiomyopathy was discovered [36]. 

Exceedingly rarely, patients might present myasthenic symptoms [37,38,39,40], although these symptoms might be disregarded in already severely muscularly impaired patients [39]. To date, phenotype–genotype correlations are still missing for the development of myasthenia in EBS-MD-affected individuals.

Patients with *PLEC* mutations might solely have skin fragility. A family with skin blistering since childhood was described by Gostýnska et al., with lesions healing with hyperkeratosis and pruritus, as well as pachyonychia [17]. The mucous membranes were unaffected, as were the teeth. No extracutaneous involvement was identified. The disease was caused by a specific homozygous nonsense mutation in the sequence of the P1a isoform, resulting in the absence of only this specific plectin isoform [17].

Finally, an autosomal dominantly inherited EBS subtype exists, also known as EBS Ogna, with mutations within the rod domain. These missense mutations are considered as changing the plectin dimer formation, leading to the proteolytic degradation of the molecule [41]. Patients only suffer from mild skin fragility—primarily on the shins and other mechanically exposed areas—mucosal erosions, and nail dystrophy. Often, violaceous plaques are found on the affected skin areas, as well as hypopigmentation [19]. Features of muscular involvement were not identified in any of the published cases [15,41,42,43]. The use of specific antibodies targeting the rod domain of plectin is helpful for the diagnosis of the disease since the staining with these is negative in the skin of individuals with EBS Ogna, while staining for antibodies recognizing other domains remains positive [42,43].

Although the clinical relevance is still elusive, mice missing the plectin 1 isoform showed diminished T-cell migration and adherence, as well as a reduced infiltration of macrophages and T-cells during wound healing [44]. Thus, further studies are required to address the relevance of plectin for wound healing and the function of the immune system.

**Table 1:** Disorders associated with plectin deficiency.

Subtype	Plectin Isotype Affected	Mode of Inheritance	Organs Affected	Mouse Model
EBS-PA	1	AR	Skin, mucosa, pylorus, urinary tract (muscle)	[45], die 2–3 days after birth
EBS-MD	1	AR	Skin, mucosa, muscle, enamel, laryngotracheal and gastrointestinal	[45], died 2–3 days after birth
Limb girdle muscular dystrophy type 2Q	1f	AR	Muscle	[46]
EBS-MD with myasthenic symptoms	1	AR	Muscle	NA
EBS Ogna	rod domain	AD	Skin	[45]
EBS-plectin 1a	1a	AR	Skin	[17]

EBS-MD, epidermolysis bullosa simplex with muscular dystrophy; EBS-PA, epidermolysis bullosa simplex with pyloric atresia; NA, not available

## 3. Plectin in Autoimmune Skin Fragility Disorders

Autoantibodies against plakins have been associated with autoimmune blistering disorders, specifically with paraneoplastic pemphigus (PNP), a life-threatening and rare disease, but also cases of more common bullous pemphigoid (BP) [47,48]. Due to its cytoplasmatic localization, pathogenicity, and development of anti-plectin antibodies, it is not fully understood, and the detection of plectin by blotting or immunoprecipitation is difficult due to its large size of 500 kDa [3]. Anti-plectin reactivity in BP, an acquired subepidermal blistering disease with autoreactivity to mainly hemidesmosomal proteins BP180 and/or BP230 [49], is a rare phenomenon and only rarely reported, while it is considered to occur through epitope spreading [50,51,52]. An analysis of plectin subdomains suggested the rod domain as being the immunodominant epitope [48].

Clinical hallmarks of PNP are polymorphous erosions, crusts, blisters, and/or lichenoid lesions with chronic and treatment-refractory mucositis, cheilitis, and conjunctivitis [50]. In addition, the disease shows an association with solid tumors or hematologic neoplasms (especially stomach, lung, or colon cancer, thymomas, and B-cell lymphomas) [53]. In 2001, the systemic nature of PNP was discussed by characterizing the multiorgan involvement occurring in patients, including respiratory or muscular manifestations, and the term “paraneoplastic autoimmune multiorgan syndrome” was introduced [51]. Direct immunofluorescence shows epithelial cell surface depositions of IgG and C3, but in around 50% of cases linear C3 also exists along the basement membrane zone of patients’ skin [52]. Indirect immunofluorescence on a monkey esophagus reveals IgG cell surface deposition, as occurring in pemphigus vulgaris or pemphigus foliaceus [50]. Several antigens have been associated with PNP. In the majority of patients, these include, besides plectin, the plakin family member envoplakin, desmoplakin I and II, desmogleins, desmocollins, BP230, and anti-alpha-2-macroglobulin-like 1 [54,55,56,57].

## 4. The Role of Plectin in Fibroblast Organization

The role of plectin in skin fibroblasts and its relevance for cutaneous disorders has remained largely unexplored. Plectin is connected to vimentin and plays a role in its network distribution within fibroblasts during the early stages of filament assembly [4]. It affects the mitosis, cell shape, polarization, and size of the cells. It also participates in forming connections between fibroblasts and their underlying extracellular matrix, while plectin-deficient fibroblasts have fewer protrusions and are rounder [58]. Their mechanical properties also seem to be impaired [59], since they are stiffer, seem to migrate less [44], and are more susceptible to stress-induced disruption [60].

## 5. Treatment Option for Patients with EBS-MD

Currently, no treatment options exist for plectinopathies, besides the symptomatic use of specific bandages for skin lesions and the early identification and management of disease-associated complications, e.g., surgical correction of the pyloric atresia. An elegant way to ameliorate the muscular phenotype due to the desmin-positive protein aggregates was described by Winter et al. with the use of the repurposed drug 4-phenylbutyrate [32]. An attempt to use this chaperone to rescue the aggregate formation and adhesion defects in keratinocytes and 3D skin equivalents of patients with EBS due to mutations in the keratin 5 or 14 genes, with a similar disease pathogenesis, was published recently [61]. However, treatment was challenging since the rescue of the aggregate formation was not followed by more stable adhesion structures, and the drug was toxic for keratinocytes in higher dosages [61]. Using other chaperones or small molecules as a symptom-relief therapy would be an interesting, symptom-relief treatment approach since gene therapies [62] or other curative approaches will be quite demanding to establish for such a rare disease and huge molecule. 

## 6. Future Perspectives

Identifying several distinct disease subtypes linked to mutations in the *PLEC* gene provided insights into the role of plectin in specific organs, especially of the different isotypes. Nonetheless, there are still open questions, e.g., regarding the muscular disease and how its progression can be influenced, and the need for an explanation of the myasthenic symptoms and the urological abnormalities that only occur in some patients. Initial data on the pathogenetic background of the disease features have been unraveled but need to be further complemented to address the role of plectin as a signaling molecule. More research into understanding the disease variability in patients with the same genetic background is also needed. Finally, novel therapeutic strategies are to be expected since several mouse models are available, as well as cell lines and 3D skin equivalents, to test possible molecules.
